# Supplementary material for: The Cost-Effectiveness of Homecare Services for Adults and Older Adults: A Systematic Review
Source: Int J Environ Res Public Health. 2023 Feb 15;20(4):3373. doi: 10.3390/ijerph20043373 (PMC9960182; doi:10.3390/ijerph20043373)
Supplement: Supplementary file 1 [file ijerph-20-03373-s001.zip › Supplementary material - Table S3.pdf]

Table S3. Quality appraisal of included studies

Study ID	Birnie 1997 [26]	Coast 1998 [20]	Cohen 2020 [16]	Cox 2018 [19]	Goossens 2013 [24]	Jafary 2020 [29]	Jones 1999 [21]	Kalra 2005 [17]	Patel 2008 [27]	Ricauda 2005 [28]	Shepperd 1998 [22]	Singh 2022 [23]	Taylor 2007 [18]	Van den Biggelaar 2020 [25]
1. Is the study population clearly described?	1	1	1	1	1	1	1	1	1	1	1	1	1	1
2. Are competing alternatives clearly described?	1	1	1	1	1	1	1	1	1	1	1	1	1	1
3. Is a well-defined research question posed in answerable form?	1	1	1	1	1	1	0	1	1	1	1	1	1	1
4. Is the economic study design appropriate to the stated objective?	0	1	1	1	1	1	1	1	0	1	1	1	1	1
5. Is the chosen time horizon appropriate in order to include relevant costs and consequences?	1	1	1	1	0	1	0	1	1	0	1	1	1	1
6. Is the actual perspective chosen appropriate?	0	1	1	1	1	1	0	1	1	0	1	1	0	1
7. Are all important and relevant costs for each alternative identified?	1	1	1	1	1	1	1	1	1	1	1	1	1	1
8. Are all costs measured appropriately in physical units?	0	1	1	1	1	1	1	1	1	1	0	1	1	1
9. Are costs valued appropriately?	1	1	1	1	1	1	1	1	0	0	1	1	1	1
10. Are all important and relevant outcomes for each alternative identified?	1	1	1	1	1	1	1	1	1	1	1	1	1	1
11. Are all outcomes measured appropriately?	1	1	1	1	1	1	1	1	1	1	1	1	1	1
12. Are outcomes valued appropriately?	0	0	0	1	1	0	0	1	1	0	0	1	0	0
13. Is an incremental analysis of costs and outcomes of alternatives performed?	0	0	1	1	1	1	0	1	0	0	0	1	1	0
14. Are all future costs and outcomes discounted appropriately?	0	1	1	0	0	0	0	1	0	0	1	1	1	1
15. Are all important variables, whose values are uncertain, appropriately subjected to sensitivity analysis?	0	1	0	0	1	1	0	1	1	0	1	1	1	0
16. Do the conclusions follow from the data reported?	1	1	1	1	1	1	1	1	1	1	1	1	1	1
17. Does the study discuss the generalizability of the results to other settings and patient/client groups?	1	1	0	1	0	1	1	1	1	0	1	1	0	1
18. Does the article indicate that there is no potential conflict of interest of study researcher(s) and funder(s)?	1	0	1	1	1	1	0	1	1	1	1	1	1	1
19. Are ethical and distributional issues discussed appropriately?	1	0	1	1	1	1	1	1	1	1	1	1	1	1
Percentage of YES	63.2%	79%	84.2%	89.5%	84.2%	89.5%	57.9%	100%	79%	57.9%	84.2%	100%	84.2%	84.2%
