# Supplementary material for: The Cost-Effectiveness of Homecare Services for Adults and Older Adults: A Systematic Review
Source: Int J Environ Res Public Health. 2023 Feb 15;20(4):3373. doi: 10.3390/ijerph20043373 (PMC9960182; doi:10.3390/ijerph20043373)
Supplement: Supplementary file 1 [file ijerph-20-03373-s001.zip › Supplementary Material Table S2. Search_Strategy.pdf]

Table S2. Search Strategy

Medline via Ovid.

1. exp Home Care Services/
2. exp Home Care Agencies/
3. exp Home Health Aides/
4. exp Respite Care/
5. exp Insurance, Long-Term Care/
6. exp Homemaker Services/
7. Home Care*.ti,ab.
8. Home-Care*.ti,ab.
9. Domiciliary Care*.ti,ab.
10. Home Health Care.ti,ab.
11. Home HealthCare.ti,ab.
12. home support.ti,ab.
13. home rehab*.ti,ab.
14. home nursing.ti,ab.
15. home based care.ti,ab.
16. home based nursing.ti,ab.
17. home based rehab*.ti,ab.
18. home based physical therapy.ti,ab.
19. home based physiotherap*.ti,ab.
20. home based occupational therapy.ti,ab.
21. Home Health Aide*.ti,ab.
22. Respite Care.ti,ab.
23. Homemaker services.ti,ab.
24. hospital at home.ti,ab.
25. home hospital.ti,ab.
26. or/1-25
27. exp Economics, Medical/
28. exp Economics, Hospital/
29. economic evaluation*.ti,ab.
30. cost effectiveness.ti,ab.
31. cost utility.ti,ab.
32. cost benefit.ti,ab.
33. (Cost* adj2 benefit*).ti,ab.
34. (Benefit* adj2 cost*).ti,ab.
35. Incremental cost*.ti,ab.
36. ICER.ti,ab.
37. Incremental Net Benefit.ti,ab.
38. INB.ti,ab.
39. Disability Adjusted Life Years.ti,ab.
40. DALY.ti,ab.
41. exp Quality-adjusted life years/
42. Quality Adjusted Life Years.ti,ab.
43. QALY.ti,ab.
44. exp Cost Analysis/
45. exp cost of illness/
46. exp Health expenditures/
47. (cost? adj2 (illness or disease or sickness)).ti,ab.
48. (burden? adj2 (illness or disease? or condition? or economic*)).ti,ab.
49. (out-of-pocket adj2 (payment? or expenditure? or cost? or spending or expense?)).ti,ab.
50. (expenditure? adj3 (health or direct or indirect)).ti,ab.
51. ((adjusted or quality-adjusted) adj2 year?).ti,ab.
52. or/27-51
53. exp hospitalization/
54. exp hospital/

55. (hospital* or conventional care or conventional management or conventional therap* or conventional treatment* or usual care or Patient Admission).ti,ab.
56. or/53-55
57. randomized controlled trial.pt.
58. controlled clinical trial.pt.
59. randomized.ab.
60. randomised.ab.
61. placebo.ab.
62. drug therapy.fs.
63. randomly.ab.
64. trial.ab.
65. groups.ab.
66. 57 or 58 or 59 or 60 or 61 or 62 or 63 or 64 or 65
67. exp animals/ not humans.sh.
68. 66 not 67
69. 26 and 52 and 56 and 68

Embase

#1 'home care services'/exp OR 'home care services' OR 'home care'/exp OR 'home care' OR 'respite care'/exp OR 'respite care' OR 'long term care insurance'/exp OR 'long term care insurance' OR 'home care*':ti,ab OR 'home-care*':ti,ab OR 'domiciliary care*':ti,ab OR 'home health care':ti,ab OR 'home-health care':ti,ab OR 'home healthcare':ti,ab OR 'home support':ti,ab OR 'home-support':ti,ab OR 'home rehab*':ti,ab OR 'home-rehab*':ti,ab OR 'home nursing':ti,ab OR 'home-nursing':ti,ab OR 'home based care':ti,ab OR 'home-based care':ti,ab OR 'home based nursing':ti,ab OR 'home-based nursing':ti,ab OR 'home based rehab*':ti,ab OR 'home-based rehab*':ti,ab OR 'home based physical therapy':ti,ab OR 'home-based physical therapy':ti,ab OR 'home based physiotherap*':ti,ab OR 'home-based physiotherap*':ti,ab OR 'home based occupational therapy':ti,ab OR 'home-based occupational therapy':ti,ab OR 'home health aide*':ti,ab OR 'respite care':ti,ab OR 'homemaker service*':ti,ab OR 'hospital at home':ti,ab OR 'home hospital':ti,ab OR 'home-hospital':ti,ab

#2 'economics, medical'/exp OR 'economics, hospital'/exp OR 'economic evaluation*':ti,ab OR 'cost effectiveness':ti,ab OR 'cost utility':ti,ab OR 'cost-effectiveness':ti,ab OR 'cost-utility':ti,ab OR ((benefit* NEAR/2 cost*):ti,ab) OR 'incremental cost*':ti,ab OR icer:ti,ab OR 'incremental net benefit':ti,ab OR inb:ti,ab OR 'disability adjusted life years':ti,ab OR daly:ti,ab OR 'quality-adjusted life years'/exp OR 'quality adjusted life years':ti,ab OR qaly:ti,ab OR 'cost analysis'/exp OR 'cost of illness'/exp OR 'health expenditures'/exp OR ((cost? NEAR/2 illness):ti,ab) OR ((cost? NEAR/2 disease):ti,ab) OR ((cost? NEAR/2 sickness):ti,ab) OR ((burden? NEAR/2 illness):ti,ab) OR ((burden? NEAR/2 disease):ti,ab) OR ((burden? NEAR/2 condition):ti,ab) OR ((burden? NEAR/2 economic*):ti,ab) OR (('out-of-pocket' NEAR/2 payment?):ti,ab) OR (('out-of-pocket' NEAR/2 expenditure?):ti,ab) OR (('out-of-pocket' NEAR/2 cost?):ti,ab) OR (('out-of-pocket' NEAR/2 spending):ti,ab) OR (('out-of-pocket' NEAR/2 expense?):ti,ab) OR ((expenditure? NEAR/3 indirect):ti,ab) OR ((expenditure? NEAR/3 direct):ti,ab) OR ((expenditure? NEAR/3 health):ti,ab) OR ((adjusted NEAR/2 year?):ti,ab) OR (('quality-adjusted' NEAR/2 year?):ti,ab)

#3 'patient admission':ti,ab OR 'usual treatment':ti,ab OR 'usual care':ti,ab OR 'conventional treatment*':ti,ab OR 'conventional therap*':ti,ab OR 'conventional management':ti,ab OR 'conventional care':ti,ab OR 'hospital*':ti,ab OR 'hospital'/exp OR 'hospitalization'/exp

#4 #1 AND #2 AND #3

#5 random*:ti,ab OR 'randomization'/de OR 'intermethod comparison'/de OR placebo:ti,ab OR compare:ti OR compared:ti OR comparison:ti OR ((evaluated:ab OR evaluate:ab OR evaluating:ab OR assessed:ab OR assess:ab) AND (compare:ab OR compared:ab OR comparing:ab OR comparison:ab)) OR (((double OR single OR doubly OR singly) NEXT/1 (blind OR blinded OR blindly)):ti,ab) OR ((open NEXT/1 label):ti,ab) OR 'double blind procedure'/de OR ((parallel NEXT/1 group*):ti,ab) OR crossover:ti,ab OR 'cross over':ti,ab OR (((assign* OR match OR matched OR allocation) NEAR/5 (alternate OR group OR groups OR intervention OR interventions OR patient OR patients OR subject OR subjects OR participant OR participants)):ti,ab) OR assigned:ti,ab OR allocated:ti,ab OR ((controlled NEAR/7 (study OR design OR trial)):ti,ab) OR volunteer:ti,ab OR volunteers:ti,ab OR 'human experiment'/de OR trial:ti

#6 'controlled clinical study'/de OR 'randomized controlled trial'/de

#7 #5 NOT #6

#8 ((random* NEXT/1 sampl* NEAR/7 ('cross section*' OR questionnaire* OR survey OR surveys OR database OR databases)):ti,ab) NOT ('comparative study'/de OR 'controlled study'/de OR 'randomised controlled':ti,ab OR 'randomized controlled':ti,ab OR 'randomly assigned':ti,ab)

#9 'cross-sectional study'/de NOT ('randomized controlled trial'/de OR 'controlled clinical study'/de OR 'controlled study'/de OR 'randomised controlled':ti,ab OR 'randomized controlled':ti,ab OR 'control group':ti,ab OR 'control groups':ti,ab)

#10 'case control*':ti,ab AND random*:ti,ab NOT ('randomised controlled':ti,ab OR 'randomized controlled':ti,ab)

#11 'systematic review':ti NOT (trial:ti OR study:ti)

#12 nonrandom*:ti,ab NOT random*:ti,ab

#13 'random field*':ti,ab

#14 ('random cluster' NEAR/3 sampl*):ti,ab

#15 review:ab AND review:it NOT trial:ti

#16 'we searched':ab AND (review:ti OR review:it)

#17 'update review':ab

#18 (databases NEAR/4 searched):ab

#19 (rat:ti OR rats:ti OR mouse:ti OR mice:ti OR swine:ti OR porcine:ti OR murine:ti OR sheep:ti OR lambs:ti OR pigs:ti OR piglets:ti OR rabbit:ti OR rabbits:ti OR cat:ti OR cats:ti OR dog:ti OR dogs:ti OR cattle:ti OR bovine:ti OR monkey:ti OR monkeys:ti OR trout:ti OR marmoset*:ti) AND 'animal experiment'/de

#20 'animal experiment'/de NOT ('human experiment'/de OR 'human'/de)

#21 #8 OR #9 OR #10 OR #11 OR #12 OR #13 OR #14 OR #15 OR #16 OR #17 OR #18 OR #19 OR #20

#22 #7 NOT #21

#23 #4 AND #22

#24 #23 NOT [medline]/lim

Web of Science

1 (TS=(“home care*” OR 'home-care*' OR “respite care” OR “long term care insurance” OR “domiciliary care*” OR “home health care” OR “home-health care” OR “home healthcare” OR “home support” OR “home-support” OR “home rehab*” OR “home-rehab*” OR “home nursing” OR “home-nursing” OR “home based care” OR “home-based care” OR “home based nursing” OR “home-based nursing” OR “home based rehab*” OR “home-based rehab*” OR “home based physical therapy” OR “home-based physical therapy” OR “home based physiotherap*” OR “home-based physiotherap*” OR “home based occupational therapy” OR “home-based occupational therapy” OR “home health aide*” OR “homemaker service*” OR “hospital at home” OR “home hospital” OR “home-hospital”))

2 ((TS=(“economic evaluation*” OR “cost effectiveness” OR “cost utility” OR “cost-effectiveness” OR “cost-utility”)) OR (TS=(benefit* NEAR/2 cost*)) OR (TS=(“incremental cost*” OR ICER OR “incremental net benefit” OR INB OR “disability adjusted life years” OR “disability-adjusted life years” OR DALY OR “quality-adjusted life years” OR “quality adjusted life years” OR QALY OR “cost analysis” OR “health expenditures”)) OR (TS=(cost? NEAR/2 (illness OR disease OR sickness))) OR (TS=(burden? NEAR/2 (illness OR disease OR condition OR economic*))) OR (TS=(“out-of-pocket” NEAR/2 (payment? OR expenditure? OR cost? OR spending OR expense?))) OR (TS=(expenditure? NEAR/3 (indirect OR direct OR health))) OR (TS=((adjusted OR “quality-adjusted”) NEAR/2 year?)))

3 TS=(“patient admission” OR “usual treatment” OR “usual care” OR “conventional treatment*” OR “conventional therap*” OR “conventional management” OR “conventional care” OR hospital*)

4 #3 AND #2 AND #1

5 TS=(randomised OR randomized OR randomisation OR randomization OR placebo* OR (random* AND (allocat* OR assign*)) OR (blind* AND (single OR double OR treble OR triple)))

6 #4 AND #5

Scopus

(((TITLE-ABS-KEY ("home care*" OR "home-care*" OR "respite care" OR "long term care insurance" OR "domiciliary care*" OR "home health care" OR "home-health care" OR "home healthcare" OR "home support" OR "home-support" OR "home rehab*" OR "home-rehab*" OR "home nursing" OR "home-nursing" OR "home based care" OR "home-based care" OR "home based nursing" OR "home-based nursing" OR "home based rehab*" OR "home-based rehab*" OR "home based physical therapy" OR "home-based physical therapy" OR "home based physiotherap*" OR "home-based physiotherap*" OR "home based occupational therapy" OR "home-based occupational therapy" OR "home health aide*" OR "homemaker service*" OR "hospital at home" OR "home hospital" OR "home-hospital")) AND (((((TITLE-ABS-KEY ((burden* W/2 (illness OR disease OR condition OR economic*))))) OR ((TITLE-ABS-KEY (("economic evaluation*" OR "cost effectiveness" OR "cost utility" OR "cost-effectiveness" OR "cost-utility" OR "incremental cost*" OR icer OR "incremental net benefit" OR inb OR "disability adjusted life years" OR "disability-adjusted life years" OR daly OR "quality-adjusted life years" OR "quality adjusted life years" OR qaly OR "cost analysis" OR "health expenditures") OR (benefit* W/2 cost*))) OR (TITLE-ABS-KEY ((cost* W/2 (illness OR disease OR sickness)))))) OR ((TITLE-ABS-KEY (("out-of-pocket" W/2 (payment? OR expenditure? OR cost? OR spending OR expense?)))))) OR ((TITLE-ABS-KEY ((expenditure? W/3 (indirect OR direct OR health))))) OR ((TITLE-ABS-KEY ((adjusted OR "quality-adjusted") W/2 year?)))) AND (TITLE-ABS-KEY (("patient admission" OR "usual treatment" OR "usual care" OR "conventional treatment*" OR "conventional therap*" OR "conventional management" OR "conventional care" OR hospital*)))) AND (((TITLE-ABS-

KEY ((randomised OR randomized OR randomisation OR randomization OR placebo*))) OR (TITLE-ABS-KEY ((random* AND (allocat* OR assign*)))) OR (TITLE-ABS-KEY ((blind* AND (single OR double OR treble OR triple))))) AND NOT ((TITLE ((rat* OR mouse OR mice OR swine OR porcine OR murine OR sheep OR lambs OR pig* OR rabbit* OR cat* OR dog* OR cattle OR bovine OR monkey* OR trout OR marmoset))))) AND NOT INDEX (medline))

CINAHL

1 MJ “home care” or MJ “home care services” OR MJ “respite care” OR TI “home care*” OR AB “home care*” OR TI “domiciliary care*” OR AB “domiciliary care*” TI “home health care” OR AB “home-health care” OR TI “home healthcare” OR AB “home healthcare” OR TI “home support” OR AB “home support” OR TI “home rehab*” OR AB “home rehab*” OR TI “home nursing” OR AB “home nursing” OR AB “home based care” OR AB “home based care” OR TI “home based nursing” OR AB “home based nursing” OR TI “home based rehab*” OR AB “home based rehab*” OR TI “home based physical therapy” OR AB “home based physical therapy” OR TI “home based physiotherap*” OR AB “home-based physiotherap*” OR TI “home based occupational therapy” OR AB “home based occupational therapy” OR TI “home health aide*” AB “home health aide*” OR TI ”respite care” OR AB ”respite care” OR TI “homemaker service*” OR AB “homemaker service*” OR TI “hospital at home” OR AB “hospital at home” OR TI “home hospital” OR AB “home hospital”
2 MJ medical economics OR MJ hospital economics OR TI “economic evaluation*” OR AB “economic evaluation*” OR TI “cost effectiveness” OR AB “cost effectiveness” OR TI “cost utility” OR AB “cost utility” OR TI (benefit* W/2 cost*) OR AB (benefit* W/2 cost*) OR TI “incremental cost*” OR AB “incremental cost*” OR TI ICER OR AB ICER OR TI “incremental net benefit” OR AB “incremental net benefit” OR TI INB OR AB INB OR TI “disability adjusted life years” OR AB “disability adjusted life years” OR TI DALY OR AB DALY OR MJ “quality adjusted life years” OR TI “quality adjusted life years” OR AB “quality adjusted life years” OR TI QALY OR AB QALY OR MJ “cost analysis” OR MJ “cost of illness” OR MJ “health expenditures” OR TI (cost? W/2 illness) OR AB (cost? W/2 illness) OR TI (cost? W/2 disease) OR TI (cost? W/2 sickness) OR TI (burden? W/2 illness) OR TI (burden? W/2 disease) OR AB (cost? W/2 disease) OR AB (cost? W/2 sickness) OR AB (burden? W/2 illness) OR AB (burden? W/2 disease) OR TI (burden* W/2 condition) OR AB (burden* W/2 condition)OR TI (burden* NEAR/2 economic*) OR AB (burden* NEAR/2 economic*) OR TI (“out-of-pocket” W/2 payment*) OR AB (“out-of-pocket” W/2 payment*) OR TI (“out-of-pocket” W/2 expenditure*) OR AB (“out-of-pocket” W/2 expenditure*) OR TI (“out-of-pocket” W/2 cost*) OR AB (“out-of-pocket” W/2 cost*)OR TI (“out-of-pocket” W/2 spending) OR AB (“out-of-pocket” W/2 spending) OR TI (“out-of-pocket” W/2 expense*) OR AB (“out-of-pocket” W/2 expense*) OR TI (expenditure? W/3 indirect) OR AB (expenditure? W/3 indirect) OR TI (expenditure? W/3 direct) OR AB (expenditure? W/3 direct) OR TI (expenditure? W/3 health) OR AB (expenditure? W/3 health) OR TI (adjusted W/2 year?) OR AB (adjusted W/2 year?) OR TI (“quality-adjusted” W/2 year?) OR AB (“quality-adjusted” W/2 year?)
3 (randomized controlled trials OR MH double-blind studies OR MH single-blind studies OR MH random assignment OR MH pretest-posttest design OR MH cluster sample OR TI (randomised OR randomized) OR AB (random*) OR TI (trial) OR (MH (sample size) AND AB (assigned OR allocated OR control)) OR MH (placebos) OR PT (randomized controlled trial) OR AB (control W5 group) OR MH (crossover design) OR MH (comparative studies) OR AB (cluster W3 RCT)) NOT ((MH animals+ OR MH animal studies OR TI animal model*) NOT MH human)
4 1 AND 2 AND 3

CENTRAL

#1 MeSH descriptor: [Home Care Services] explode all trees
#2 MeSH descriptor: [Respite Care] explode all trees
#3 MeSH descriptor: [Home Care Agencies] explode all trees
#4 MeSH descriptor: [Home Health Aides] explode all trees
#5 MeSH descriptor: [Insurance, Long-Term Care] explode all trees
#6 MeSH descriptor: [Homemaker Services] explode all trees
#7 (“home care*” OR “domiciliary care*” OR “home health care” OR “home healthcare” OR “home support” OR “home rehab*” OR “home nursing” OR “home based care” OR “home based nursing” OR “home based rehab*” OR “home based physical therapy” OR “home based physiotherap*” OR “home based occupational therapy” OR “home health aide*” OR “respite care” OR “homemaker service*” OR “hospital at home” OR “home hospital”):ti,ab,kw
#8 #1 or #2 or #3 or #4 or #5 or #6 or #7
#9 MeSH descriptor: [Economics, Medical] explode all trees
#10 MeSH descriptor: [Economics, Hospital] explode all trees
#11 MeSH descriptor: [Quality-Adjusted Life Years] explode all trees
#12 MeSH descriptor: [Costs and Cost Analysis] explode all trees
#13 MeSH descriptor: [Cost of Illness] explode all trees
#14 MeSH descriptor: [Health Expenditures] explode all trees
#15 MeSH descriptor: [Cost of Illness] explode all trees

#16 (“economic evaluation*” OR “cost effectiveness” OR “incremental net benefit” OR INB OR “disability adjusted life years” OR DALY OR “quality adjusted life years” OR QALY OR “cost analysis” OR “health expenditures” OR “incremental cost*” OR ICER):ti,ab,kw (Word variations have been searched)
#17 (benefit* NEAR/2 cost*):ti,ab,kw (Word variations have been searched)
#18 (cost? NEAR/2 (illness OR disease OR sickness)):ti,ab,kw
#19 (burden? NEAR/2 (illness OR disease OR condition OR economic*)):ti,ab,kw
#20 (“out-of-pocket” NEAR/2 (payment? OR expenditure? OR cost? OR spending OR expense?)):ti,ab,kw (Word variations have been searched) 89524
#21 (expenditure? NEAR/3 (indirect OR direct OR health)):ti,ab,kw (#22 ((adjusted OR “quality-adjusted”) NEAR/2 year?):ti,ab,kw
#23 #9 or #10 or #11 or #12 or #13 or #14 or #15 or #16 or #17 or #18 or #19 or #20 or #21 or #22
#24 #8 and #23
#25 MeSH descriptor: [Hospitalization] explode all trees
#26 MeSH descriptor: [Hospitals] explode all trees
#27 (“patient admission” OR “usual treatment” OR “usual care” OR “conventional treatment*” OR “conventional therap*” OR “conventional management” OR “conventional care” OR hospital*):ti,ab,kw (Word variations have been searched)
#28 #25 or #26 or #27
#29 #24 and #28
